# Integration Strategies in the Implementation of a Cardiology Chronic Care Clinic in a Primary Care Setting: A Qualitative Study

**DOI:** 10.5334/ijic.10197

**Published:** 2026-09-03

**Authors:** Shilpa Surendran, Rakhi Vashishtha, Brigitte Fong Yeong Woo, Ash Yusi Zhang, David Bruce Matchar

**Affiliations:** 1 Department of Health Services and Systems Research, Duke-NUS Medical School, 8 College Rd, Singapore; 2 Centre for Behavioural and Implementation Science Interventions, Yong Loo Lin School of Medicine, National University of Singapore, Singapore; 3 Department of Alice Lee Centre for Nursing Studies, National University of Singapore, 10 Medical Dr, Singapore; 4 Department of Medicine (General Internal Medicine), Duke University School of Medicine, 40 Duke Medicine Circle, 124 Davison Building, Durham, United States of America

**Keywords:** integrated care, integration, primary health care, chronic disease, qualitative research

## Abstract

**Introduction:**

A Cardiology Integrated Chronic Care (ICC) clinic was established at a polyclinic through collaboration between a tertiary hospital and a polyclinic. This novel model in Singapore prompted a study exploring explore how stakeholders conceptualise and operationalise integration when implementing the clinic in primary care, aiming to understand if common cardiology conditions can be anchored in primary care.

**Methods:**

Nine semi-structured interviews were conducted with a cardiologist, intervention’s director, family physician, polyclinic head, coordinators and advanced practice nurse. Twelve documents —including implementation monitoring reports, steering committee reports and meeting minutes — were analysed using Singer and colleagues’ conceptual model of integration types.

**Results:**

Co-located consultation rooms, direct access investigations and remote consultations between healthcare providers promoted structural integration. Interpersonal integration was fostered through teamwork. Functional integration was accomplished via comprehensive care management protocols and training. Low patient numbers reduced polyclinic revenue but positively impacted care efficiency. Provider satisfaction remained high, with participants gaining knowledge and confidence in managing cases.

**Conclusion:**

The clinic demonstrated operational integration but may lack a broader healthcare system perspective. For sustainable larger-scale evolution, economic and policy scrutiny is needed focusing on operational feasibility and outcomes, given low patient volume constraints affecting financial viability and scalability potential.

## Introduction

Cardiovascular diseases account for about a third of global deaths, with ischemic heart disease (IHD) and atrial fibrillation (AF) among the most prevalent conditions [1,2]. Studies have identified several barriers to managing IHD and AF, including lack of consensus among physicians on management strategies, difficulties ensuring consistent follow-up care, and poor patient adherence to treatment [3]. Suboptimal management of IHD and AF can strain healthcare systems through increased hospitalisations, clinic visits, emergency department admissions, avoidable procedures, and related costs [4]. To improve management of IHD and AF, the European Society of Cardiology has recommended integrated care approaches [5].

Care integration is the process of harmonising separate components of healthcare systems to create a synergistic effect across activities within and between them [6]. It is advocated for several reasons [7]. First, it focuses on the patient and their needs rather than on diseases, organ systems, and procedures. Second, it supports care tailored to individuals with diverse needs by facilitating coordination among the multiple actors involved in holistic care. Third, by creating a locus of accountability, it can reduce duplication of services across medical specialities.

Integrated chronic care (ICC) models for cardiovascular conditions have been studied mainly in hospital- and specialty-based settings, with nurse-led AF clinics being the most common configuration [8,9]. In recent years, internationally and in Singapore, there has been a growing trend to anchor chronic disease management of common conditions in primary care. This shift reflects the view that primary care is the natural locus of care coordination, has lower outpatient unit costs than hospital-based specialty care, and, with appropriate support, can achieve outcomes comparable to specialty providers [10,11]. Recognising this opportunity, particularly in the context of Healthier SG, a national effort to strengthen primary care, many integrated care efforts have been introduced in primary care in Singapore [12,13,14,15,16,17]. One recent example is the Cardiology ICC clinic within the National University Hospital System, one of Singapore’s three regional healthcare clusters [14].

The Cardiology ICC clinic is a collaboration between Bukit Batok (BBK) Polyclinic and the Cardiology Department of National University Hospital (NUH) [14]. A polyclinic is a one-stop healthcare centre that provides primary medical treatment, preventive healthcare and health education [18]. This Cardiology ICC clinic was established to manage patients with IHD and AF. These conditions were chosen for ICC because patients benefit from close collaboration through a multi-disciplinary team approach.

Limiting the Cardiology ICC clinic to common cardiac issues also reflected recognition that primary and specialty care, in Singapore and more broadly, have evolved distinct organisational cultures. Integration across these cultures is not self-evident, and the challenges would likely increase as the number of specialty links expanded.

The translation of integrated care models into primary care settings remains underexplored. In Singapore specifically, while several primary care initiatives have been documented no study has qualitatively examined how integration is conceptualised and operationalised when cardiology services are anchored in a primary care setting in Singapore [19]. This gap is significant for three reasons. First, it leaves healthcare providers and managers without evidence-based guidance on the practical strategies needed to achieve integration across specialist-primary care boundaries. Second, it limits policymakers’ ability to anticipate the operational, financial, and cultural challenges that such models will encounter. Third, it obscures the conditions under which integration efforts are likely to succeed or fail at scale. Therefore, the research question we sought to answer was: How do stakeholders conceptualise and operationalise integration when implementing a Cardiology ICC clinic in primary care? This study addresses this gap by providing a detailed, stakeholder-informed account of how integration was built, in one such clinic — offering insights for healthcare providers, managers, and policymakers involved in establishing any specialty-focused ICC clinics within primary care settings [20]. To our knowledge, no Cardiology ICC clinic has been implemented in a primary care setting in Singapore.

## Methods

### Intervention: Cardiology ICC clinic

The Cardiology ICC clinic started in September 2021 as a pilot at BBK polyclinic to reduce avoidable referrals of patients with AF and IHD to cardiology outpatient clinics at NUH. It is primarily managed by five family physicians, two advanced practice nurses (APNs), and two care coordinators. An APN is a registered nurse who has completed the Master of Nursing Programme [21]. A cardiologist from NUH supports the clinic remotely. The clinic operates only on Fridays. It is considered an active intervention because, compared with usual care, it integrates services, increases touchpoints and monitoring, and modifies operational and direct care elements. The clinic has since become permanent.

### Changes in operation for care pathway

In usual care, BBK polyclinic lacks a specific pathway for managing AF and IHD. Typically, family physicians refer patients presenting at the general pool with AF or IHD symptoms to the cardiology specialist outpatient clinic (SOC) at NUH, where cardiologists manage their care. In contrast, the Cardiology ICC clinic establishes defined care pathways. Patients first seen in the general pool are referred to the Cardiology ICC clinic if they have AF or IHD symptoms, such as atypical chest pain, shortness of breath, reduced effort tolerance, or abnormal ECG/scan findings requiring further investigation, and if they need to switch to non-vitamin K antagonist anticoagulant. The wait time to see a family physician is 1–2 weeks. During this time, care coordinators prepare pre-consultation notes and disseminate them to the team. If necessary, the family physician orders specialised tests and refers the patient to NUH, with a wait time of about 6–8 weeks. Depending on the results, patients with normal findings are discharged back to the general pool at BBK polyclinic, while those with abnormal findings may be referred to NUH.

**Figure 1:**
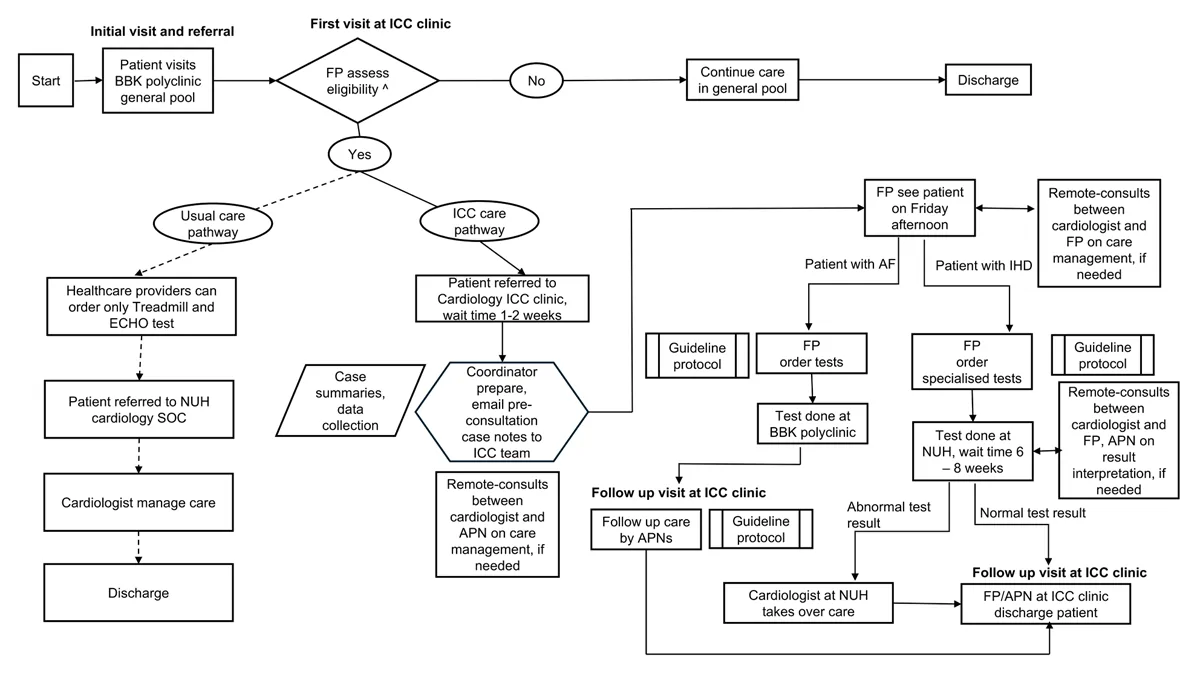
Workflow process of Cardiology ICC clinic. AF = atrial fibrillation, APN = advance practice nurse, BBK = bukit batok polyclinic, FP = family physician, Cardiology ICC = integrated chronic care, IHD = ischemic heart disease, NUH = national university hospital, SOC = specialist outpatient clinic, ^Eligibility criteria includes patients with suspected IHD symptom e,g., atypical chest pain, shortness of breath and reduced effort tolerance, abnormal ECG/scan for further investigation (may not have symptom), newly diagnosed AF and existing AF for discussion switch to non-vitamin K antagonist anticoagulant.

In usual care, healthcare providers at BBK polyclinic could order only Treadmill and Echo tests. With the Cardiology ICC clinic, they can also order more specialised tests, such as myocardial perfusion imaging and CT coronary angiograms, which were previously ordered only by cardiologists at SOC. In addition, family physicians and APNs can communicate directly with cardiologists via messages or email, unlike in usual practice. The cardiologist also interprets diagnostic test results, such as CT coronary angiograms and myocardial perfusion scans, for family physicians or APNs via teleconsultation when needed. The detailed workflow of the clinic is presented below in Figure 1.

### Conceptual framework

We used Singer et al.’s conceptual framework because the Cardiology ICC clinic represents an active intervention where there is integration of services, increased patient-healthcare provider touch points, active monitoring, and modifications in operation and direct care elements [6]. Typically, healthcare service integration occurs through structural, functional, and clinical types of integration. However, the social side of integration is equally important, as positive social relationships between team members are crucial for achieving effective integration. Singer and colleagues have outlined structural, functional, and clinical types of integration, and incorporated normative and interpersonal integration as the social aspects of integration [6].

### Study design

We used qualitative research methods, incorporating two data collection techniques: i) in-depth semi-structured interviews and ii) document review. This combination provided multiple sources of evidence to capture the integration efforts. Participants were not involved in the study design.

### Sampling strategy and data collection

In-depth semi-structured interviews (IDIs): Data were collected between March and October 2023 using an interview guide available in the appendix. The interview guide was developed based on a review of the relevant literature [6,20,22,23,24]. Using convenience sampling, we recruited stakeholders who were involved in the planning, development, and implementation of the Cardiology ICC Clinic. Convenience sampling was chosen because the eligible participant pool was small and well-defined. We obtained a contact list from the programme manager and sent potential participants the participant information sheet and consent form via email. All participants approached agreed to participate. Participants included a cardiologist, intervention’s director, two coordinators, two APNs, a family physician, and the head of BBK polyclinic. IDIs were conducted after obtaining written informed consent. The first author (SS) conducted all IDIs in English, each lasting 60 to 90 minutes.

### Document review

The first author (SS) reviewed twelve documents: the clinic’s implementation monitoring reports, steering committee reports and meeting minutes. These documents were provided by the intervention team. We treated the meeting minutes as authentic because they included attendance records, were signed by the intervention’s director and were part of the institution’s official records. The documents were complete and contained both positive and negative aspects of the intervention.

## Data analysis

IDIs: IDIs were audio-recorded, transcribed verbatim and analysed using Lumivero NVivo 14 software deductively using Singer et al.’s framework [6,25]. Authors (SS) and (DM) led the analysis and organised the codes into sub-themes and themes.

To enhance the rigour of the analytical process, we discussed codes and themes in regular team meetings to foster reflexivity and challenge interpretations. Thematic saturation was reached at the 9th interview when no new codes or themes were identified [26]. Data collection therefore stopped after the 9th interview. Additionally, according to Guest et al. thematic saturation is reached after six to 12 interviews [27]. Quotes are identified by a participant label (P) followed by a unique participant number (e.g., P01).

To enhance the trustworthiness of the study, we implemented several measures. Firstly, the interviewer transparently acknowledged her role as a research team member to the participants, thereby mitigating any potential preconceived bias [28]. Secondly, the interviewer wrote memos to record her reflections and capture emerging themes, which were subsequently used to characterise the various types of integration. Lastly, member checking was performed with selected participants to confirm the accurate representation of their perspectives and validate our interpretations of the data [29].

Document analysis: In the preliminary phases of the study, the documents provided basic knowledge about the Cardiology ICC Clinic and the types of resources and materials available. Similar to the IDI analysis, documents were analysed by authors (SS) and (DM) deductively using Singer et al.’s framework in Lumivero NVivo 14 [6,25].

Data integration: Codes from transcripts were compared with document codes to identify similarities, differences and general patterns. Data from both sources were given equal weightage and merged at the analysis stage by (SS) using Singer et al.’s conceptual framework [6].

### Positionality

The interviewer is a medical doctor (non-practising) with formal university training in qualitative research methods. The interviewer was able to build rapport and trust with the participants and interpret the data with greater nuance because of the shared healthcare background. Nevertheless, the interviewer was aware that the familiarity with the healthcare system while an asset in contextual interpretation, could also predispose her to certain assumptions. To mitigate this, interpretations were discussed with the broader research team regularly to ensure that findings remained grounded in participants’ own perspectives rather than the interviewer’s prior knowledge or beliefs.

## Results

Nine IDIs were conducted with a cardiologist, intervention’s director, two coordinators, two APNs, a family physician, and the head of BBK polyclinic. Three participants were male and six were female. Themes and subthemes are described below. Table 1 summarises the findings and the authors’ practice recommendations. To indicate the prevalence of themes, we reported the number and percentage of participants expressing particular views. These figures are presented descriptively to show the distribution of perspectives while preserving the qualitative nature of the thematic analysis.

**Table 1:** Integration types, summary of findings and practice recommendations.


THEME	DESCRIPTION ACCORDING TO SINGER AND COLLEAGUES	SUMMARY OF FINDINGS (STRATEGIES THAT HELPED TO ACHIEVE CARDIOLOGY ICC CLINIC’S INTEGRATION IN BUKIT BATOK POLYCLINIC)	PRACTICE RECOMMENDATION TO ACHIEVE INTEGRATION WHEN IMPLEMENTING A CARDIOLOGY ICC CLINIC IN PRIMARY CARE

**Structural Integration**	“Physical, operational, financial, or legal ties among teams and organisations in a health system” (p.201)	Team members interested in managing cardiac conditions nominated by senior staff members	Involve senior staff of the primary care centre in nominating team members to ensure team’s commitment and expertise. This can include creating a selection committee and establishing clear criteria for selection to ensure a motivated and skilled team

		Co-location of consultation rooms enhanced collaboration in case management	Co-location of consultation rooms of APNs and family physicians in primary care centre. Review and adjust the physical layout of the clinic to ensure it continues to meet the changing needs and workflows of the team

		Remote case consultations by cardiologist via emails and messages facilitated by care coordinators	Team to develop standardised protocols for care coordinators’ pre-consultation case preparation and information dissemination, including templates for case descriptions and checklists to ensure all relevant information is included and communicated efficiently. Integrate these protocols into the electronic medical record system

**Functional Integration**	“Formal, written policies and protocols for activities that coordinate and support accountability and decision making among organisations and individuals” (p.201)	Comprehensive written protocols for AF and IHD management developed in collaboration by cardiologist, APNs and family physicians	Team to develop comprehensive written protocols for management of cardiac conditions in collaboration

		e-learning programs and on-the-job training by cardiologist supervising family physicians at Cardiology ICC clinic and APNs and family physicians as observers in cardiology clinics at National University Hospital	Formalise e-learning programs with dedicated time and incentives, such as paid leave, professional development credits and potential salary increments upon certification completion. This investment in continuous education can significantly enhance the competency and confidence of the healthcare team

**Normative Integration**	“Sharing a common culture and exhibiting a culture that prioritises integrating patient care across units and organisations within a health system” (p.201)	Team shared a vision to anchor chronic disease management in primary care	To develop and sustain a shared vision that effectively anchors chronic disease management in primary care. This can include creating a clear, concise vision statement that reflects the shared goals for chronic disease management in primary care and embedding the vision into the clinic’s standard operating procedures and protocols

**Interpersonal Integration**	“Collaboration or teamwork among health care professionals, nonprofessional caregivers, and patients” (p.201)	Introducing to patients the team, communicating the clinic’s purpose, roles of team members and clinic’s operational days	Each team member to introduces themselves personally to the patient and explain how each will contribute to patient’s care. This can be done during the first consultation. Use name badges with roles clearly indicated

**Process Integration**	“Courses of organisational actions or activities intended to integrate patient care services into a single coordinated process across people, functions, and operating units over time” (p.201)	Closed-loop referrals	Develop standardised referral protocols that clearly outline the steps for initiating, managing, and closing referrals

**Outcomes of care integration**	Not applicable	Low patient numbers at Cardiology ICC clinic Family physicians outside Cardiology ICC clinic forgetting to refer patients to ICC clinic Less patient footfall on clinic’s operation day	Develop a targeted communication strategy to raise awareness about the Cardiology ICC clinic among family physicians. This can include direct communication through electronic medical records reminders to encourage referrals To reevaluate and potentially adjusting the operating hours of the clinic by testing alternative days and times that might be more convenient for patients, such as midweek or evenings instead of Fridays

		Less revenue generated from the Cardiology ICC clinic Mismatch between consultation time and fees charged	Explore alternative revenue model such as capitated funding model instead of fee for service model (currently used in Singapore) which can better capture the value of comprehensive and integrated care provided at the Cardiology ICC clinic

		Provider satisfaction Increased knowledge and confidence for APNs and family physicians in managing patients with AF and IHD	Provide access to accredited continuous medical education courses specifically targeting cardiac conditions of interest Establish mentorship programmes where experienced cardiologists mentor APNs and family physicians, offering guidance, support and feedback when in managing cardiac conditions of interest


AF: Atrial fibrillation, APN: Advanced practice nurse, Cardiology ICC: Cardiology Integrated chronic care, IHD: Ischemic heart disease.

### Theme 1: Structural integration

The Cardiology ICC clinic achieved structural integration by forming a clinical care team comprising APNs, family physicians, and care coordinators. According to five participants (5/9, 56%), senior staff members nominated individuals interested in managing cardiac conditions to join the team. They also highlighted that family physicians could directly order investigations at NUH without needing to refer patients to the cardiologist. Additionally, the clinic strategically designed its physical layout, positioning consultation rooms for APNs and family physicians in close proximity to enhance collaboration in case management. One participant mentioned:

“We wanted to co-locate APN and family physician so that they can consult each other.” (P09)

The clinic also developed structural integration between family physicians and the cardiologist through remote case consultations facilitated by messages and emails. Five participants (5/9, 56%) reported that care coordinators played a pivotal role in this process. Coordinators regularly disseminated detailed case descriptions for the upcoming week to family physicians and cardiologists so that family physicians could readily access expert opinions from the cardiologist when they sought them. One participant mentioned:

“We (coordinators) prepare cases in advance… email family physicians and cardiologist so that they can prepare.” (P01, 02)

### Theme 2: Functional integration

The team achieved functional integration in many ways. All participants mentioned that, first, the cardiologist, APNs, and family physicians collaboratively developed formal, distinct, comprehensive written protocols for AF and IHD management. The protocols encompassed treatment targets, recommended medications, laboratory investigations, echocardiogram procedures, and the timing and frequency of these assessments. One participant mentioned:

“…the protocol spells out more on how frequently should we do certain labs,…routine labs,…what medications…. is very helpful” (P07)

On the contrary, two participants (2/9, 22%) stated that APNs held limited medication prescribing authority. According to them, APNs could draft prescriptions but required a family physician to approve (sign off) them. However, for most cases (80%) they indicated a high level of confidence in prescribing medications for patients. The participants emphasised that delegating prescribing authority to APNs could yield time-saving benefits for family physicians. One participant mentioned:

“we cannot (prescribe) for AF clinic…we draft the medicine, and then the doctor need to approve it… I am confident to prescribe most of the medications of AF for pts (80%) of cases… save the doctor a bit of time.” (P08)

Second, many participants mentioned undergoing on-the-job training and e-learning courses to help them manage patients with AF and IHD. Five participants (5/9, 56%) mentioned that the team conducted on-the-job training in two ways. Initially, the cardiologist supervised the family physicians on Friday afternoons at the Cardiology ICC clinic. Later, APNs and family physicians visited NUH to observe the cardiologist managing the cardiology clinics. New family physicians joining the Cardiology ICC clinic also underwent on-the-job training by sitting in as observers of the already trained family physicians in the Cardiology ICC clinics. In tandem with on-the-job training, APNs pursued an 8-week, 40-hour certified e-learning course for managing patients with AF. However, one participant (1/9, 11%) mentioned that despite completing the certification course, APNs did not receive a salary increase. Moreover, the clinic did not grant APNs leave to pursue the e-learning course, requiring them to allocate personal time for completion.

Another crucial aspect of functional integration involved the cardiologist providing feedback on performance indicators to the family physicians. Some performance indicators included the number of direct access investigations ordered, the number of SOC visits avoided, and the monthly utilisation rate of the Cardiology ICC clinic. This feedback mechanism reinforced family physicians’ capability to function independently. The document analysis revealed that between July 2022 and June 2023, family physicians and APNs achieved utilisation rates of 77.4% and 53.5%, respectively, at the Cardiology ICC clinic.

### Theme 3: Normative integration

The team shared a vision to anchor chronic disease management in primary care. To achieve this, they aimed to expand the clinic’s scope to encompass additional specialties. However, most participants (7/9, 78%) mentioned, and document analysis revealed, that specialities such as ophthalmology requiring significant capital outlay in the form of equipment cost might not suit the integrated care model.

The team also acknowledged limitations in the types of cases that APNs could manage when integrating other specialities. Despite the urgency to anchor patients in primary care, one participant (1/9, 11%) raised concern about potentially diluting the scope of the polyclinic by developing integrated speciality clinics within it. One participant mentioned:

“I don’t think it’s sustainable. We can’t have different clinic for every discipline…not polyclinic anymore. I think this clinic really blur line between primary care and tertiary care… by (and) large the bread and butter is primary care management.” (P09)

### Theme 4: Interpersonal integration

Interpersonal integration was achieved through teamwork. Two participants (2/9, 22%) reported that the family physicians introduced the team, communicated the purpose of the clinic and the distinct roles of each team member, operational days, and the availability of remote consultations between the cardiologist and family physicians and APNs to the patients. Additionally, APNs actively engaged in elucidating their additional roles as nurse educators in care management. They also mentioned the cardiologist conducting talks and reminding all family physicians at BBK polyclinic about the Cardiology ICC clinic. Furthermore, during consultations, coordinators strategically positioned themselves to facilitate seamless data collection, proactively gathering granular data without explicit directives. One participant mentioned:

“…family physician will introduce us to the patients. He will tell the clinic’s purpose, members of this clinic, explaining this clinic will always be fixed on this day…” (P07)

All participants also praised the cardiologist for his prompt responsiveness, approachability, encouragement, and non-judgmental character when they contacted him through remote consultations.

### Theme 5: Process integration

The team achieved process integration through closed-loop referrals and an interoperable electronic information system. All participants mentioned, and document analysis showed, that after stabilising the condition, the clinic discharged 66.6% and 78% of AF and IHD cases, respectively, back to referral sources. Moreover, family physicians could access the results of direct access investigations done at NUH.

### Theme 6: Outcomes of care integration

#### Subtheme 6.1: Low patient numbers at Cardiology ICC clinic

All participants echoed low patient numbers as a challenge, and document analysis revealed that between June 2021 and June 2023, the clinic saw only 39 patients with AF and 198 patients with IHD. Participants cited patients’ preferences to seek care at other centres and the forgetfulness of family physicians outside the Cardiology ICC clinic to refer patients as some of the reasons. According to one participant (1/9, 11%), the choice of Friday for operating the clinic appeared to negatively influence patient attendance because patients prioritised fulfilling personal commitments over seeking care. One participant mentioned:

“…Friday afternoon, most people probably want to gather somewhere. So even if we go and see other clinic, they also have less patient compared with other afternoons.” (P08)

#### Subtheme 6.2: Efficiency of care

Low patient numbers positively impacted care efficiency, as most participants (6/9, 67%) indicated and document analysis confirmed. This brought several advantages. Firstly, patients experienced a brief waiting period of 16 days for consultation appointments and gained direct access to investigations. However, increased patient numbers could extend appointment wait times for direct access investigations due to limited overall appointment availability. Secondly, the cardiologist did not need to redistribute his typical workload among colleagues, enabling him to handle both remote consultations with family physicians to discuss the management of patients in the Cardiology ICC clinic and his regular SOCs. Thirdly, the cardiologist could promptly schedule appointments for patients with abnormal test results at the cardiology SOC within a week due to the reduced patient volume. One participant mentioned:

“…I tend to give them a very early date (within a week) and it’s very individual driven process… because the numbers are overall small, I’ve kept it this way…” (P06)

#### Subtheme 6.3: Less revenue for BBK polyclinic

According to some participants (4/9, 44%), the Cardiology ICC clinic generated less revenue for the polyclinic. This was primarily due to the low patient numbers. For example, in the afternoon, only seven patients were seen by family physicians at the Cardiology ICC clinic, while 15 patients received care outside this setting. Despite a seemingly modest difference in patient numbers, this contrast could lead to a substantial loss when calculated annually. Another contributing factor to reduced revenue was the extended consultation duration, taking around 30 minutes for patients with AF or IHD. However, they were billed for only 20 minutes, the standard charge for patients with chronic conditions, resulting in a mismatch between service time and fees charged. One participant mentioned:

“…on a Friday afternoon my doctor will end up seeing 7 cardio patients versus 15 regular patients. So 50 percent cut in terms of revenue from the patient…” (P04)

“…we are charging for 20 minutes, but we actually using 30 minutes or even more to see one AF case.” (P13)

#### Subtheme: 6.4 Provider satisfaction

Most participants (7/9, 78%) expressed provider satisfaction. They reported gaining extensive knowledge and growing confidence in handling patients with AF and IHD through functional integration. Document analysis revealed that the referral rates of these patients to the specialist outpatient clinic decreased by 80%. APNs were observed to practice at the peak of their licensure by effectively managing the care of these specific patient cohorts. One participant mentioned:

“…APNs practice at the top of their license…they achieved that and…ability to manage reasonably complex chronic patients.” (P05)

## Discussion

Our study suggests that participants operationalised integration through a series of organised strategies. Co-location of consultation rooms, remote case consultations and availability of direct access investigations promoted structural integration. Interpersonal integration was fostered through teamwork. Functional integration was accomplished via comprehensive care management protocols and training. Low patient numbers at the Cardiology ICC clinic and less revenue from the Cardiology ICC clinic were significant challenges. However, low patient numbers positively impacted care efficiency. Moreover, positive provider satisfaction was evident, as participants gained extensive knowledge and confidence in managing these conditions.

The low patient volume at the Cardiology ICC clinic could be due to patients’ care-seeking habits. In Singapore, patients have the freedom to seek care from any healthcare institution islandwide. Patients may also hold fixed ideas about which types of medical conditions can be managed in primary care versus tertiary care.

The low patient volume likely had a dual and paradoxical effect on integration. On one hand, it facilitated integration by reducing the operational pressure on the team. This created conditions for functional integration to take root. On the other hand, low volume constrained the integration effort by limiting the number of cases through which the team could build collective clinical competence and refine protocols. It also reduced the political and economic visibility of the clinic, making it harder to justify continued investment or expansion. Future research should examine the policy determinants of this low-volume phenomenon which is beyond the scope of this study.

The elements of structural integration within the Cardiology ICC clinic, such as the co-location of team members, were strategically achieved through the design of the clinic’s physical layout. This approach is consistent with findings from other studies that emphasise the importance of spatial organisation in enhancing communication and teamwork among healthcare providers [30,31]. However, a noteworthy observation was that family physicians not managing the Cardiology ICC clinic sometimes failed to refer patients to the Cardiology ICC clinic. This may be linked to the clinic’s spatial separation from regular clinics located on a different floor. The physical distance can create a disconnect, making it less likely for family physicians not managing the Cardiology ICC clinic to think of the Cardiology ICC clinic as an immediate resource for their patients. Therefore, physical placement of integrated clinics within a facility should be treated as a deliberate integration strategy, not merely a logistical decision. Furthermore, operating the clinic on Friday afternoon should be treated as a modifiable structural variable. Stakeholders must consider piloting alternative operating days and tracking referral rates and patient attendance before committing to a fixed schedule.

Additionally, maintaining interpersonal connections between family physicians operating in the Cardiology ICC clinic and those overseeing the regular clinics could be challenging. This challenge partly stems from the discrepancy in patient volumes, as family physicians at the Cardiology ICC clinic tend to see fewer patients compared to their counterparts in the regular clinics. This discrepancy in workload perception can inadvertently spark feelings of discontent and lead to a perception that Cardiology ICC clinic’s family physicians have more available time. If unaddressed, it can risk undermining the normative integration that underpins team cohesion. Despite these challenges, the role of care coordinators in disseminating case information is consistent with existing literature on coordinated care, which highlights the need for a central figure to streamline communication and keep team members informed and engaged in patient management [32].

On the functional integration front, the development of formal, comprehensive written protocols mirrors findings from other studies [20]. Moreover, the delegation of prescribing authority to APNs, although limited, aligns with studies advocating for task-shifting to optimise physician time and improve workflow efficiency [33]. Functional integration was also achieved partly by externalising costs onto individual providers. This is not a sustainable foundation for integration at scale. It suggests that the clinic’s functional integration was, in part, contingent on the goodwill and personal sacrifice of its staff – a form of what might be termed integration by attrition. If staff turnover occurs, the competence base built through this informal training arrangement may not be readily replaceable. Finally, providing performance feedback is comparable to other studies that stress its importance enhancing healthcare providers’ competency [34].

On the process integration front, high discharge rate reflects an intentional design choice to keep the Cardiology ICC clinic as a transitional, rather than a permanent, locus of care. Interpreted through an integration lens, this suggests the clinic operationalises integration not as the indefinite retention of patients within a specialised stream, but as a capacity-building exercise for the broader primary care system stabilising patients and returning them to general primary care, thereby expanding the overall system’s capacity to manage cardiac conditions.

These strategies can be replicated in primary care centres planning to implement integrated chronic care clinics. The current momentum favouring chronic disease management within primary care settings has sparked the implementation of numerous interventions [12,13,14]. However, the simultaneous deployment of multiple primary care initiatives and expansion to other polyclinics invites resource allocation challenges. Such challenges are inherently unavoidable due to the finite nature of healthcare provider capacities and facility availability. In this resource-constrained environment, it becomes critical that all primary care initiatives including ICC models undergo careful scrutiny of their scalability before broader rollout, as models demonstrating efficacy in a single setting may not translate effectively at scale. Navigating these resource constraints necessitates focusing on optimising interventions for the collective benefit of the population [35]. Consequently, interventions must undergo economic and policy analysis, emphasising operational practicality and outcomes that contribute to the aggregate good and exert significant social impact [35].

### Comparison with other models internationally

Our findings resonate with a growing international body of evidence on integrated cardiology-primary care models. Across the Spanish models [36,37], the Dutch Support Consultation [38], the Barcelona heart failure programme [39], the preventive cardiology integration review [40], and the multidisciplinary AF care review [41], a consistent theme emerges: structured specialist-primary care linkages whether through embedded cardiologists, formalised consultation pathways, or defined care protocols improve guideline adherence, reduce unnecessary referrals, and enhance provider satisfaction. Our clinic shares these broad structural features, particularly the use of defined care protocols, closed-loop referral systems, and deliberate mechanisms for specialist-primary care communication. However, important differences exist. The Spanish and Dutch models physically embedded cardiologists within primary care centres, whereas our clinic achieved integration through remote consultations and pre-consultation case preparation by coordinators suggesting that physical co-location, while beneficial, may not be essential. The specific use of APNs as the primary clinical contact is also distinctive to our model and not a feature of any of these international comparators, representing a more pronounced form of task-shifting to non-physician providers. The Comín-Colet et al [39] programme demonstrated population-level reductions in morbidity and mortality at a scale our clinic has not yet reached, underscoring that integration must be accompanied by sufficient patient volume to generate measurable outcomes. Alaujan et al [40] highlighted pharmacists as key integration enablers, a role absent in our model and worth considering in future iterations. Collectively, these studies and our own findings suggest that sustainable integrated cardiology care in primary care requires not only structural and functional integration mechanisms, but also deliberate attention to workforce capacity, referral pathway maintenance, and economic feasibility before broader scale-up is pursued.

### Steps ahead: Economic and policy scrutiny to assess scalability of the integrated care model

Whether the Cardiology ICC clinic is cost-saving for the healthcare system as a whole remains an empirical question that this study was not designed to answer. When we develop integrated care models of care delivery, we must be cautions against assuming that integration automatically reduces costs. In many cases, costs are shifted rather than eliminated. For example, from hospital-based SOC to primary care infrastructure, staff training, and coordination overhead. In the case of this clinic, the reduced referral rate to the SOC (80% reduction) may represent avoided costs at the tertiary level, but these must be weighed against the costs incurred at the polyclinic, including extended consultation times, care coordinator roles, and APN training to arrive at a meaningful net cost estimate. A formal economic evaluation, such as a cost-effectiveness or cost-consequence analysis, is therefore essential before broader rollout is considered. Ex-ante economic modelling rather than post-hoc evaluation should be used to test financial viability.

Policy scrutiny is also necessary because our findings indicate that the integrated chronic care clinic in the primary care setting potentially blurs the line between primary care and specialty care. This shift may confuse both patients and healthcare providers about the primary care centre’s core mission. It could also potentially divert resources away from primary care, undermining its overall effectiveness. Additionally, it could confuse patients about where to seek care and healthcare providers about their roles. Therefore, careful economic and policy scrutiny is essential before scaling this service.

### Programme implications

First, a dedicated care coordinator role must be resourced from the outset. Second, formal on-the-job training with protected time must be built into the programme design. The current arrangement, in which APNs completed 40-hour certification courses in their personal time without salary recognition, might not be sustainable. Third, the operating schedule must be empirically tested. The Friday afternoon slot was associated with lower patient attendance, and programme managers should pilot alternative days before committing to a fixed schedule. Fourth, referral pathways must be actively maintained through electronic medical record reminders and periodic refreshers to family physicians outside the ICC team, given evidence that referral rates dropped when the clinic was not top of mind.

### Strength and limitations

The study involved the meticulous collection of comprehensive data, employing multiple methods. Data triangulation bolstered the credibility of the study findings, ensuring they closely mirrored the actual circumstances surrounding the development of integration. Nevertheless, it must be acknowledged that because this is a cross-sectional study, it did not capture the clinic’s evolution over time. We learned that changes occurred, but their explicit details were not clear to us. Therefore, we could not explicitly delineate how these changes might have impacted the integration process. Future research employing longitudinal designs would be valuable in tracking the development and evolution of integration strategies as the clinic matures. Furthermore, there is a possibility the intervention team may have provided us with only a selected subset of documents, a situation that could introduce a bias known as ‘biased selectivity’ [42]. This might have limited the depth of insights derived from our analytical approach, constraining our comprehensive understanding of the integration process and its dynamics.

While this study is situated within Singapore’s healthcare system, the integration mechanisms identified are not inherently Singapore-specific and can inform implementation efforts in other contexts. For example, co-location of clinics, closed-loop referrals, task-shifting to APNs, remote specialist consultation, and comprehensive protocol development are strategies that can be actively pursued across many healthcare systems internationally. These mechanisms are actionable across health systems with varying levels of resources, provided that workforce scope-of-practice regulations, financing models, and referral pathway infrastructure are adapted to local conditions. Health systems considering similar integrated chronic care clinics should treat these five mechanisms as a foundational implementation checklist, while subjecting their local financial and policy environment to appropriate scrutiny.

## Conclusion

The Cardiology ICC clinic has demonstrated commendable multi-faceted integration, as indicated by the findings. However, these findings also highlight a crucial point: while the integration is commendable within its context, it may fall short in the broader healthcare system perspective. For the intervention to evolve into a sustainable, larger-scale service, it necessitates economic and policy scrutiny, emphasising operational feasibility and outcomes that benefit the collective well-being and hold substantial social influence. This is particularly significant given the clinic’s low patient numbers.

## Additional File

The additional file for this article can be found as follows:

10.5334/ijic.10197.s1 Appendix. Interview guide.

## Data Availability

The datasets generated during and/or analysed during the current study are not publicly available due to participants’ privacy concerns. Readers who wish to gain access to the data can write to the corresponding author; data may be granted upon reasonable request.
