# Supplementary material for: Integration Strategies in the Implementation of a Cardiology Chronic Care Clinic in a Primary Care Setting: A Qualitative Study
Source: Int J Integr Care. 2026 Sep 3;26(3):13. doi: 10.5334/ijic.10197 (PMC13544038; doi:10.5334/ijic.10197)
Supplement: Supplementary file 1 [file ijic-26-3-10197-s1.pdf]

Interview guide

General questions

1. What is your occupation?
2. How long have you had this role?
3. What is your role in the intervention?

Perceptions of actors

1. Who are the different actors involved in the intervention?
2. How do the different actors participate in the intervention?

Facilitators – for developing the ICC clinic

1. Which factors facilitated the development of the intervention? [prompts: location, training, relationship with cardiologist, protocols, access to tests in hospital etc]
2. Elaborate each of the prompts?
3. Any other facilitators?
4. What sort of changes/practices/requirements were needed?

Barriers – to developing the ICC clinic

1. Which factors hindered the development of the intervention? [patient numbers, revenue etc]
2. Elaborate how?
3. Any other barriers?

Relationships

1. How are the roles of the actors clearly defined?
2. How is the information about the intervention communicated between the different actors involved?
3. What have you gained?

Nature of the intervention

1. How is the intervention conducted? Elaborate
2. What strategies were used?
3. What happens when not resolved?
4. How is it interpreted?
5. How are resources managed and used for the intervention?
6. Did the actors receive feedback?
7. Can you give an example of the feedback?
8. What happens when you receive the feedback?

NUS Confidential
